# Human brucellosis in pregnancy – An overview

**DOI:** 10.17305/bjbms.2019.4499

**Published:** 2020-11

**Authors:** Mile Bosilkovski, Jurica Arapović, Fariba Keramat

**Affiliations:** 1 University Clinic for Infectious Diseases and Febrile Conditions, Medical Faculty Skopje, Skopje, Republic of North Macedonia; 2 Working Group on Zoonoses, International Society for Chemotherapy, Aberdeen, United Kingdom; 3 Brucellosis Research Center, Hamadan University of Medical Sciences, Hamadan, Iran; 4 Department of Infectious Diseases, University Clinical Hospital Mostar, Mostar, Bosnia and Herzegovina; 5 Faculty of Medicine, University of Mostar, Mostar, Bosnia and Herzegovina

**Keywords:** Seroprevalence, brucellosis, pregnancy, complications, treatment

## Abstract

Human brucellosis during pregnancy is characterized by significantly less pronounced adverse obstetric outcomes than in animals, but with remarkably more adverse obstetric outcomes when compared to healthy pregnant women. Seroprevalence of brucellosis in pregnancy and cumulative incidence of brucellosis cases per 1000 delivered obstetrical discharges in endemic regions were reported to be 1.5–12.2% and 0.42–3.3, respectively. Depending on the region, the frequency of pregnant women in the cohorts of patients with brucellosis was from 1.5% to 16.9%. The most common and the most dramatic unfavorable outcomes during brucellosis in pregnancy are the obstetric ones, manifested as abortions (2.5–54.5%), intrauterine fetal death (0–20.6%), or preterm deliveries (1.2–28.6%), depending on the stage of pregnancy. Other unfavorable outcomes due to brucellosis are addressed to infant (congenital/neonatal brucellosis, low birth weight, development delay, or even death), the clinical course of disease in mother, and delivery team exposure. When diagnosed in pregnant women, brucellosis should be treated as soon as possible. Early administration of adequate therapy significantly reduces the frequency of adverse outcomes. Rifampicin in combination with trimethoprim-sulfamethoxazole for 6 weeks is the most commonly used and recommended regimen, although monotherapies with each of these two drugs are also widely used while waiting for the results from prospective randomized therapeutic trials. As no effective human vaccine exists, screening of pregnant women and education of all women of childbearing age about brucellosis should be compulsory preventive measures in endemic regions.

## INTRODUCTION

Human brucellosis is one of the most common zoonoses in the world and important public health problem in many parts of Africa, South and Central America, Asia, and the Mediterranean region [1,2]. Clinically, it is presented as febrile disease with affection of various body systems [3] or as a fever of unknown origin [4]. The disease is contracted through direct contact with infected animals, ingestion of unpasteurized dairy products, or by aerosol inhalation [3,5,6].

Human brucellosis is ubiquitous, found in all age groups and both genders likewise [3,5,7] and, consequently, pregnant women can acquire it as well. In the absence of well-designed prospective studies, the current knowledge about brucellosis in pregnant women is based on observational studies and case reports [8]. Therefore, many important questions regarding the incidence of brucellosis in pregnancy, the effect on obstetric outcome and infant health, and vice versa, the influence of pregnancy on the severity and outcome of brucellosis remain unanswered.

The aim of this study is to assess different aspects of brucellosis in pregnancy based on the data found in the current literature.

## HISTORY

The first human abortion due to *Brucella* infection was reported in 1905 by Thierry in France, followed by Devoir in 1906 who described a case of abortion in pregnant farmer [9,10]. In 1908, Eyre recognized the occurrence of brucellosis during pregnancy [11]. In 1917, De Forest proposed a correlation between abortion and active brucellosis in humans, despite the fact that they were unable to prove it microbiologically [12]. Preterm delivery due to brucellosis was reported for the first time by De Carle in 1931 [13,14]. In 1938, Vecchio published the first case series of 59 pregnant women with brucellosis; among them, 78.6% had a spontaneous abortion [15]. The first case of congenital brucellosis was reported by Hagebusch and Frei in 1941 [16].

## PREVALENCE OF HUMAN BRUCELLOSIS IN PREGNANCY AND VICE VERSA

The incidence and prevalence of brucellosis among pregnant women is unknown even today in many endemic regions [17]. According to various reports, a seroprevalence of brucellosis during pregnancy varied between 1.5% (13 seropositive out of 890 pregnant women) [18], 3.5% (18 out of 513) in rural areas of Saudi Arabia [19], 5.8% (25 out of 429) in Pakistan [20], and 12.2% (55 out of 450) in another study from Saudi Arabia [21]. The cumulative incidence of brucellosis cases in pregnancy per 1000 delivered obstetrical discharges was estimated to be from 0.42 [22] to 3.3 [23].

In cohorts of patients with brucellosis, pregnant women comprised 19 out of 1245 (1.5%) [24] and up to 92 out of 545 cases (16.9%) [25]. In addition, Buzgan et al. reported 17 pregnant women among 1028 patients with brucellosis (1.7%) [26], Kurdoglu et al. reported 21 pregnant women out of 342 patients with brucellosis (6.1%) [22], Madkour’s study reported 30 pregnancies among 500 patients with brucellosis (6%) [27], while in the study of Glick et al. 11 out of 114 patients (9.6%) were pregnant [28]. The largest recently published multicenter study found 242 (2.1%) pregnant women among 11,602 adult brucellosis patients [23].

Having in mind that some of the above-mentioned studies were not based on universal microbiological diagnostic criteria, there is still a possibility of some minor differences in brucellosis seroprevalence (Table 1).

**TABLE 1:**
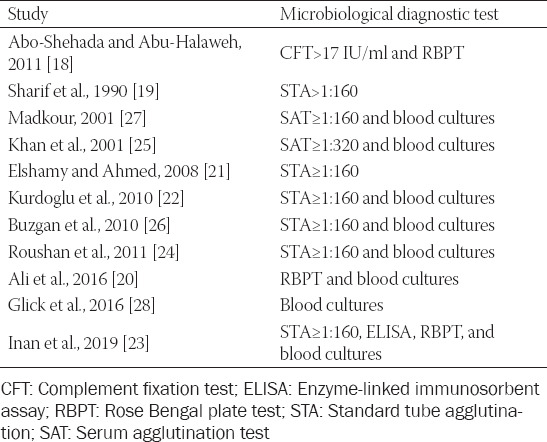
Diagnostic criteria for seroprevalence of human brucellosis in pregnancy

## THE INFLUENCE OF HUMAN BRUCELLOSIS ON OBSTETRIC OUTCOMES

Contrary to the well-known fact that *Brucella* infection in animals is associated with a high incidence of abortion, data about the relationship between the disease and pregnancy outcome in humans are controversial [29-31].

According to the previous experiences, mainly of older date, brucellosis does not play a role in the occurrence of adverse obstetric outcomes during human pregnancy [32]. Spink also did not find definitive evidence that *Brucella* species produce abortions any more frequently than other bacterial species [33]. In the same line, several newer studies from endemic regions demonstrated that *Brucella* seroprevalence among pregnant women with and without a history of spontaneous abortion was similar, i.e., the women with a spontaneous abortion were not more commonly seropositive than those with normal pregnancy outcome [18,31,34]. It is important to emphasize that, as a control group in these three studies, the prevalence of abortions among the general population was investigated instead of abortion prevalence among seronegative women.

Contrary to these findings, some contemporary data suggest that brucellosis has a significant role in adverse obstetric outcomes in humans, and they imply that *Brucella* species may indeed produce human abortions more frequently than other bacterial pathogens [25]. With the rate of adverse obstetric outcomes from 14% to 46%, brucellosis exceeds the rate that can be seen in the general population of pregnant women [13,21,23]. In the context of such assertions are positive culture isolates of *Brucella* spp. obtained from human placenta, aborted fetuses or preterm stillbirths, and other outcomes of conception [13,27,35-37]. The first large series on the causative relationship between abortion in humans and brucellosis was published by Criscuolo and Di Carlo and reported 52 abortions among 200 pregnant women with active brucellosis (26%) [38]. The authors confirmed their findings by positive blood culture of *Brucella melitensis* from maternal blood in one, maternal urine in two, and uterine tissue culture in one case [27,38]. An association between human brucellosis incidence and adverse pregnancy outcomes was also documented in a study from Israel, especially having in mind numerous sociodemographic cofactors that were applied. The rates of preterm delivery, intrauterine fetal death (IUFD), and poor fetal growth were significantly higher in Israeli-Arab localities with a high incidence of brucellosis compared to localities where the disease was not reported [39].

As previously mentioned, brucellosis is an established factor of spontaneous abortion or sterility in animals [40]. In humans, brucellosis causes fewer spontaneous abortions than in animals as a result of the absence of erythritol in the woman’s placenta [27,41,42]. Erythritol is a sugar alcohol and it is considered an important growth factor for *Brucella* spp. that can be found in large amounts in animal placentas. Furthermore, additional reasons for the potential role of brucellosis in the incidence of adverse obstetric outcomes in humans might be attributed to maternal bacteremia, disseminated intravascular coagulation (DIC), placentitis, and acute febrile reaction. Thus, released endotoxins could also be an important cause of adverse obstetric outcomes, since endotoxins increase the frequency and intensity of uterine contractions by means of an oxytocin-like effect on uterine smooth muscles [10,43,44]. Finally, allergic mechanisms in chronic brucellosis may also cause spasms of the myometrium by histamine discharge [10,17]. In addition, it has been recently observed that pathogenic *Brucella* species can proliferate in human trophoblasts and are able to interfere with the invasive capacity of extravillous trophoblasts. This is crucial for implantation during the early stages of pregnancy and could possibly play a central role during early abortion in women with brucellosis [45]. It is also noteworthy to mention that in pregnant animal models, interferon (IFN)-γ induced by the immune response plays an important role in causing abortion during brucellosis [46].

## INCIDENCE OF HUMAN BRUCELLOSIS AS A CAUSE OF ADVERSE PREGNANCY OUTCOMES

Many studies have found a significantly increased risk of abortion and IUFD in women with brucellosis compared to healthy ones (Table 2). Contrary to the study of Elshamy and Ahmed [21], which did not find a significant difference in terms of preterm delivery, in the study of Gulsun et al., it was obvious that brucellosis in pregnancy increases the incidence of preterm delivery compared to healthy pregnant women – 17.9% (7 out of 39) and 2.5% (1 out of 40), respectively [29].

**TABLE 2:**
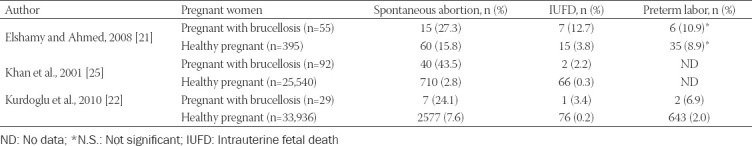
Adverse obstetric outcomes in pregnant women with and without brucellosis

In the reports originating from Kuwait, Iran, Rwanda and Nigeria, brucellosis was confirmed in 2 out of 29 (6.9%) [47], 6 out of 51 (11.8%) [35], 11 out of 60 (18.3%) [48] and 23 out of 121 (19%) [49] women that exhibited spontaneous abortion, respectively. Brucellosis was also found in 5 out of 51 (9.8%) women that manifested IUFD and in 18 out of 227 (7.9%) women with preterm delivery [47].

## TYPES OF OUTCOMES IN PREGNANT WOMEN WITH BRUCELLOSIS

As shown in Table 3, the outcomes of brucellosis in pregnancy can be observed from different aspects. Mainly, the outcomes depend on the prompt and appropriate treatment of the disease in pregnant women.

**TABLE 3:**
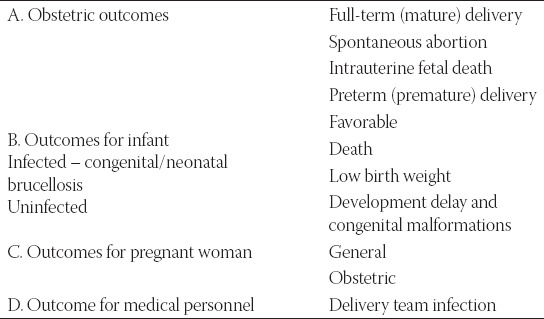
Outcomes in pregnant women with brucellosis

### Obstetric outcome

Obstetric outcomes are manifested as favorable (full-term delivery) and unfavorable (abortion, IUFD, and preterm delivery). Unfavorable obstetric outcomes were found in 34 out of 242 (14%) pregnant women suffering from brucellosis [23]. In the same study, splenomegaly, vomiting, vaginal bleeding, anemia, elevated serum aspartate aminotransferase, oligohydramnios, history of taking medication other than brucellosis treatment during pregnancy, and *Brucella* bacteremia were the significant potential risk factors for unfavorable outcome [23].

Full-term delivery in pregnant women with brucellosis ranged from 47.4% (9 out of 19) [24] to 100%, found in small series of four patients [50]; and full-term delivery was also reported in 15 out of 29 (51.7%) [27], 19 out of 29 (65.5%) [22], 21 out of 39 (53.8%) [29], 50 out of 86 (58.1%) [9], and in 219 out of 242 (90.5%) [23] pregnant women with brucellosis. In conclusion, full-term delivery is primarily associated with early recognition of brucellosis during pregnancy and adequate treatment of the disease.

In brucellosis during pregnancy, spontaneous abortion (fetal death that occurs at ≤24 weeks of gestation) is more frequent than IUFD (fetal death that occurs at >24 weeks of gestation) and preterm delivery (the birth of a baby before 37 weeks of gestation) [22]. The abortion rate was reported to be from 1 out of 39 (2.5%) [29] up to 6 out of 11 (54.5%) [51], and mainly in the range between 17.6% and 41.0% of pregnant women with brucellosis [22,27,52]. In the study by Inan et al., the abortion rate was only 6.2% (15 out of 242), which is lower than usually reported frequencies – this could be attributed to the early establishment of diagnosis and appropriate treatment [23]. Abortions were noted mostly in the first trimester [9,24,27], although other studies did not find a difference in the incidence of abortion according to the trimester [25].

The rate of IUFD ranges between 0 [29] and 20.6% [13]. It was detected in 2.1% (5 out of 242) [23], 3.4% (1 out of 29) [27], 8.1% (7 out of 86) [9], 9.1% (1 out of 11) [53], and 12.7% (7 out of 55) [21] of pregnant women with brucellosis.

Preterm delivery due to brucellosis is well recognized with rates between 1.2% (3 out of 242) [23], 9.1% (1 out of 11) [51], 14.0% (12 out of 86) [9], 17.9% (7 out of 39) [29], and up to 28.6% (2 out of 7) [30]. Also, preterm delivery was associated with congenital brucellosis as well as growth and developmental delay, and as such it is considered as a major determinant of immediate and long-term morbidity of the infant [54,55].

### Outcome for infants

Outcomes for infants are the second most important consequence of brucellosis during pregnancy. The newborn can be either uninfected, which is a more frequent outcome, or infected and characterized by congenital or neonatal brucellosis. Uninfected newborns are usually associated with full-term delivery. Congenital brucellosis can be contracted transplacentally, whereas neonatal brucellosis can be acquired through the contact with body fluids secreted during delivery or by breastfeeding in the postpartum period [43,56-58]. However, congenital brucellosis is a rare condition, most of the cases are associated with preterm delivery [55,59], and it occurs in approximately 2% of infants exposed to brucellosis *in utero* [60]. From 1988 to 2007, only 15 cases of congenital brucellosis were reported in the literature [55]. Nevertheless, in the study by Vilchez et al., 4 out of 86 (4.6%) patients had congenital brucellosis [9]. Clinical manifestations of congenital brucellosis are serious and the morbidity as well as mortality rates are high [43,61]. This condition can be clinically presented with poor feeding, fever, jaundice, respiratory distress syndrome, meconium aspiration syndrome, sepsis, and multiple organ failure [56,62-64], so it is very difficult to clinically distinguish congenital brucellosis from other bacterial infections [43]. Nevertheless, favorable outcome in congenital brucellosis was described as well [56].

Favorable outcome was evident in most of uninfected and full-term delivered newborns, whereas in preterm cases and cases with congenital brucellosis, an increased risk for neonatal death is obvious. After delivery, neonatal death occurred in 2 out of 36 infants (5.6%) from mothers who were treated for brucellosis [25] and in 7 out of 86 (8.1%) in another study [9]. Low birth weight [LBW] (<2500 g) of infants from mothers who had brucellosis during pregnancy was reported in 7% (17 out of 242) [23], 14.5% (9 out of 62) [9], and up to 25.6% (10 out of 39) [29]. The general impression is that brucellosis in pregnant mothers was not associated with congenital malformations [17,29,57,62].

### Outcome for pregnant women

The age of pregnant women with brucellosis ranged from 15 to 50 years, with the majority aged between 25 and 29 years [9,23,48]. Positive epidemiological (family) history in pregnant women who had brucellosis was 61.3% [65], 63.0% [22], and 76.9% [29]. Clinical course of human brucellosis during pregnancy was the same as the course observed in non-pregnant patients and ranged from asymptomatic to severe disease [44]. Most of the pregnant women suffered from the acute form and manifested as mild illness [9]. Clinical symptoms in pregnant women with brucellosis were non-specific, consisting of weakness, arthralgia, fever, fatigue, excessive night sweating, lack of appetite, myalgia, chills, depression, weight loss, headache, and back pain. The most common signs were fever, hepatomegaly, splenomegaly, and osteoarticular affection [23,29]. Other focal manifestations were recognized as well [22,29,54]. However, one study from Israel noticed that complications in pregnant population were present in 45%, which was significantly higher than 10% in non-pregnant women [28]. Similarly, in another study, focal brucellosis was found in 46.7% (113 out of 242) among pregnant population [23]. The most frequent laboratory finding was anemia and elevated erythrocyte sedimentation rate [29]. Gram-negative sepsis and DIC [66], as well as maternal death as a complication of severe sepsis, [9] were sporadically described in pregnant women. Relapses and chronicity can occur during pregnancy as well as in all other patients that suffer from brucellosis, although in the study performed by Inan et al., relapses were extremely rare (0.4%) [23].

Obstetric manifestations in women with brucellosis were vaginal bleeding in 9.1% (22 out of 242) [23], postpartum endometritis in 28.6% (2 out of 7) [30], groin pelvic pain in 23.5% (8 out of 34) [23], as well as preterm rupture of membranes [43,55,56] and chorioamnionitis [60,67]. Repeated abortions were also described among women with brucellosis [24,27], and one old report found infertility in 19% [10] but this was not further confirmed [24,27].

###  Outcome for medical personnel

Outcomes for the medical personnel include the exposure and possible infection of the delivery team due to contact with infective amniotic fluid, and there are several cases described so far [57,62,64].

## CORRELATION BETWEEN *BRUCELLA* ANTIBODY TITER AND HUMAN PREGNANCY OUTCOME

There are contradictory results concerning the association between pregnancy outcome and level of antibody titer or blood culture positivity. According to some authors, there is a connection between *Brucella* antibody titers ≥1:160 and spontaneous abortion. Women with titers 1:160 were twice at risk of having a spontaneous abortion as compared to those with lower titers. When the titer was higher than 1:160, the incidence of abortion was 17.6% and 44% in the Sharif [19] and the Elshamy and Ahmed [21] study respectively, whereas when the titer was less than 1:160, the incidence was 7.7% and 19%, respectively [19,21]. These findings were not confirmed in cases of IUFD and preterm delivery [21]. On the other side, other studies did not find a correlation between the *Brucella* antibody titers and spontaneous abortion [24,25,31]. Serum agglutination titers (SAT) ≥1:2560 were not significantly associated with spontaneous abortion when compared with the lower titers [25]. Also, the abortion rates in patients with SAT <1:640 and ≥1:640 were 45.5% and 62.5%, respectively, i.e., not significantly different [24].

Furthermore, there were contradictory data about the relationship between obstetrical outcomes and the presence of maternal bacteremia. In one report, abortions were registered in 8 out of 22 (36.4%) women with and in 16 out of 30 (53.3%) women without *Brucella* bacteremia, which was not statistically significant [25]. On the other hand, Garriguet et al. reported two spontaneous abortions in three bacteremic women and no abortion among 13 pregnant culture-negative women with brucellosis (*p* < 0.05) [68].

## PRINCIPLES OF BRUCELLOSIS TREATMENT DURING HUMAN PREGNANCY

Until now, no clinical trials on the treatment of brucellosis during pregnancy have been particularly conducted. The therapy in this group of patients is mostly based on expert recommendations, observational studies, case series [9] as well as on clinical experience and tradition [69]. The key points in the treatment of brucellosis in pregnancy are early recognition and prompt initiation of antimicrobial therapy as the measures that can decrease the risk of unfavorable obstetric, neonatal, maternal, and delivery team outcomes [25,29,54,57]. In one case series of 19 pregnant women, among 13 patients who received antimicrobial treatment, only 4 aborted and 9 had full-term deliveries, whereas all 6 untreated women aborted [24]. In other series of 11 pregnant women with brucellosis, 3 were adequately treated and delivered full-term infants, whereas 8 untreated women manifested adverse outcomes [51].

Therapy of brucellosis in pregnancy is still challenging, since pregnant women cannot take tetracyclines due to their potential to cause fetal tooth staining, although the risk from doxycycline is much lower in comparison to other tetracyclines [61,70]. Quinolones are also not recommended during pregnancy because of their chondrotoxicity. The administration of streptomycin or gentamicin during pregnancy poses the risk of ototoxicity or nephrotoxicity in the infant [61]. Thus, the preferred antimicrobials in pregnant women are rifampicin and trimethoprim-sulfamethoxazole (TMP-SMX). The latter is associated with neonatal kernicterus and its use is not recommended after the 36^th^ gestational week [71]. If TMP-SMX is used anyway, supplementation of folinic acid should be given [61]. Rifampicin is the safest of all available antibiotics that can be used by pregnant women with brucellosis [1].

## THERAPEUTIC COMBINATIONS IN PREGNANT WOMEN WITH BRUCELLOSIS

For the treatment of brucellosis in pregnancy, rifampicin in combination with TMP-SMX for 6 to 8 weeks is the most commonly used and preferred regimen [13,24,57], despite the findings that the incidence of abortions among 22 patients treated with TMP-SMX monotherapy was not significantly different from that of 17 patients treated with a combination of TMP-SMX and rifampicin [25]. Rifampicin is the mainstay of brucellosis treatment during pregnancy [2] and the World Health Organization advises rifampicin monotherapy as the first line [72]. Monotherapy is still questionable in case of brucellosis treatment and further randomized studies should give the answer whether this option is suitable for the treatment of pregnant women with brucellosis.

Some authors treat brucellosis in pregnancy with gentamicin for 1 week plus TMP-SMX for 6 weeks, with [9] or without [32] rifampicin. In the study of Inan et al., 11 different regimens composed of ceftriaxone, rifampicin, TMP-SMX, doxycycline, and streptomycin/gentamicin were used and no association between any of three widely used combinations (rifampicin plus TMP-SMX, rifampicin plus ceftriaxone, and rifampicin plus TMP-SMX plus ceftriaxone) and the occurrence of adverse pregnancy outcomes was found [23]. Another study with a small number of cases compared the treatment outcome of four different regimens including TMP-SMX monotherapy, rifampicin monotherapy, TMP-SMX plus rifampicin, and ceftriaxone plus rifampicin, and the overall conclusion was that the ceftriaxone-rifampicin combination therapy was the most effective one [29]. Having in mind that a significant rate of antimicrobial resistance of *Brucella* has been recently observed *in vitro* for rifampicin and TMP-SMX [73], ceftriaxone could also be a rational choice in the combination treatment approach and a promising regimen for treating pregnant women with brucellosis in endemic regions.

For neonatal brucellosis, the treatment of choice should be the combination of TMP-SMX and rifampicin for 6 weeks, or TMP-SMX for 6 weeks and gentamicin for the first week [56]. After the birth (delivery/abortion/IUFD), the treatment of woman may be switched to doxycycline and rifampicin for 6 weeks, or doxycycline for 6 weeks and streptomycin for the first 2–3 weeks or gentamicin for the first week [9]. If mothers breastfeed, it is a general opinion that breastfeeding should be discontinued until the completion of treatment. In that case, based on the previous experience, therapy with a combination of ceftriaxone and rifampicin should be a reasonable choice [29]. Also, it has been recently published by the American Academy of Pediatrics that doxycycline is a favorable drug for a maximum of 3 weeks of therapy, even in infants and children below 8 years of age [74]. Thus, the question addressed to the authorities for brucellosis treatment is whether this regiment should be reconsidered in some of combination varieties.

## PREVENTION

In the absence of an adequate vaccine for human use, non-specific measures like screening and education of pregnant women and testing of suspicious cases may help to prevent the disease and its complications during pregnancy. In endemic regions, pregnant women should be routinely tested for brucellosis [21,24,59]. Also, in those areas, women of childbearing age should be educated what brucellosis is, i.e., what the ways of acquiring the disease are, what the main clinical manifestations are, how it is diagnosed, and what the possible consequences are if left untreated [22,24]. Finally, in endemic areas, brucellosis should be considered in differential diagnosis of all pregnant women with febrile disease with/without persistence of unspecific symptoms including affection of various organs and systems. Likewise, all cases with unexplained spontaneous abortion, IUFD, preterm delivery, LBW, fetal death, or previous history for these conditions should be tested for brucellosis [9,20,24,57].

## CONCLUSION

Brucellosis can be found among pregnant women with a significant frequency in endemic regions. The incidence of adverse obstetric outcomes in women with brucellosis exceeds the rates among general population. Furthermore, brucellosis during pregnancy might have a negative influence on the newborn’s health and might cause delivery team infection. Early recognition of the disease and timely administration of antimicrobial therapy can significantly decrease the risk of unfavorable obstetric, neonatal, maternal, and delivery team outcomes. Screening and education of pregnant women as well as of all women of childbearing age should be compulsory measures to prevent the disease in endemic regions for brucellosis.
